# Tranexamic acid reduces blood loss in primary total hip arthroplasty performed using the direct anterior approach: a one-center retrospective observational study

**DOI:** 10.1186/s10195-022-00638-7

**Published:** 2022-03-07

**Authors:** Guo-Chun Zha, Xian-Ren Zhu, Lei Wang, Hong-Wei Li

**Affiliations:** 1 Department of Orthopedic Surgery, The Affiliated Hospital of Xuzhou Medical University, No. 99 Huaihai West Road, Xuzhou, 221002 Jiangsu People’s Republic of China; 2 Department of Orthopedic Surgery, Mudan People’s Hospital of Heze City, No. 2111 Kangzhuang Road, Mudan District, Heze, 274000 Shandong People’s Republic of China; 3 Department of Orthopedics, The First Affiliated Hospital of Wannan Medical College, Wuhu, 241001 Anhui People’s Republic of China

**Keywords:** Total hip arthroplasty, Tranexamic acid, Blood loss, Direct anterior approach

## Abstract

**Background:**

It is still unknown whether tranexamic acid (TXA) is beneficial for the minimally invasive surgical approach to total hip arthroplasty (THA). The aim of this study is to investigate the efficacy and safety of intravenous TXA in primary THA via the direct anterior approach (DAA).

**Materials and methods:**

We performed a retrospective analysis of prospectively collected data on 70 patients with nontraumatic avascular necrosis of the femoral head who underwent THA via the DAA between October 2017 and October 2018. Patients were divided into two groups: TXA group (39 patients received 1.5 g TXA intravenously) and control group (31 patients did not receive TXA). Patients were assessed by operative time, postoperative hemoglobin (HB) drop, transfusion rate, postoperative length of hospital stays (LHS), deep vein thrombosis (DVT), and Harris hip score (HHS).

**Results:**

Total blood loss, hidden blood loss, and postoperative HB drop in the TXA group were significantly lower than in the control group (*p* < 0.05). There was no statistical difference between the two groups in terms of intraoperative blood loss, operative time, transfusion rate, postoperative LHS, HHS, or incidence of DVT (*p* > 0.05).

**Conclusions:**

TXA may reduce perioperative blood loss without increasing complications in THA via the DAA.

**Level of evidence:**

Level IV, therapeutic study.

## Introduction

Total hip arthroplasty (THA) has been widely used for the treatment of end-stage hip disease, which can effectively relieve pain, restore function, and improve quality of life [1]. It has been reported that the total blood loss during the perioperative period of THA can be as high as 2000 mL, with a transfusion rate as high as 37% [2–4]. Massive transfusion not only increases the risk of surgery, but also causes transmission of viral diseases, hemolytic reactions, immune reactions, and other transfusion-related risks [5, 6].

With the popularization of the minimally invasive concept and the continuous improvement of prosthesis materials and design, there are a variety of THA surgical approaches available clinically, such as direct anterior approach (DAA), anterolateral approach, posterior approach, posterolateral approach, SuperPATH approach (SuperPATH approach, namely supercapsular percutaneously assisted approach, which is a direct superior portal-assisted approach for THA that utilizes the interval between the gluteus minimus and the piriformis to access the hip capsule), and lateral approach. Among them, DAA is a minimally invasive surgical approach through a natural intermuscular and internervous interval. This approach has the advantages of minimizing soft tissue disruption and reducing the incidence of dislocation [7–9]. Tranexamic acid (TXA) is a synthetic derivative of the amino acid lysine, which can reduce fibrinolysis through the reversible blockade of lysine-binding sites on plasminogen molecules [10]. As a synthetic antifibrinolytic agent, TXA has been shown to be effective in reducing blood loss and transfusion rate in THA [11, 12]. However, few studies have explored the efficacy of TXA in minimizing perioperative blood loss in primary THA with DAA [13–15]—in other words, it remains unclear whether TXA is beneficial for the minimally invasive surgical approach to THA.

Therefore, this study aimed to investigate the efficacy and safety of intravenous TXA in THA via the DAA. We hypothesized that the use of TXA would be associated with less blood loss, without increasing the rates of complications, when compared with the control in primary THA performed using the DAA.

## Materials and methods

### Patient source

We performed a retrospective analysis of prospectively collected data on 80 patients (90 hips) with nontraumatic avascular necrosis of the femoral head who underwent total hip arthroplasty (THA) via the direct anterior approach (DAA) between October 2017 and October 2018. Patients were excluded if they had the following: (1) bilateral THA (10 patients); (2) incomplete radiographic or clinical data (0 patients); (3) follow-up time less than 3 months (0 patients). After applying the exclusion criteria, 70 patients (70 hips) qualified for the study.

Patients were divided into two groups: TXA group (39 patients received 1.5 g TXA intravenously) and control group (31 patients did not receive TXA). This study was approved by the ethics committee of Affiliated Hospital of Xuzhou Medical University (no. 20170829). All methods were performed in accordance with the relevant guidelines and regulations, and all patients gave informed consent.

#### Study setting

All surgeries were performed by the senior author (Z.G.C) using cementless THA via DAA. All patients received general anesthetic and the same design of the femoral stem (CLS stem; Zimmer, Warsaw, USA) and acetabular cup (Trilogy; Zimmer, Warsaw, USA). We did not use a wound drainage after the procedure. In the TXA group, TXA was given as a 1.5 g intravenous infusion 10 min prior to incision; the control group did not receive TXA.

All patients were managed with a similar perioperative regimen, including intravenous prophylactic antibiotics, prophylaxis against venous thrombosis, and postoperative pain control.

Patients were transfused if their postoperative hemoglobin level was below 70 g/L or if the patient had a hemoglobin above 70 g/L and below 100 g/L but poor mental status, palpitation, or pale complexion. All patients underwent deep vein ultrasound of the lower limbs 1 week postoperatively to detect thrombosis.

#### Data collection

Data were collected on patient characteristics including sex, age, body mass index (BMI), preoperative hemoglobin (HB), preoperative hematocrit (HCT), and American Society of Anesthesiologists (ASA) classification. HB and HCT levels were also measured at each timepoint on postoperative days 1 and 3. Operative time, transfusion rate, postoperative HB drop, postoperative length of hospital stays (LHS), and Harris hip score (HHS) were recorded. Total blood loss and pulmonary blood volume (PBV) were calculated according to the Gross and Nadler equation [16, 17]. The discharge criteria for patients with THA in our hospital are as follows: (1) stable vital signs, (2) good mental and physical status, (3) no nausea/vomiting, (4) pain control, and (5) no redness, swelling, or exudate from the incision.

PBV = *k*_1_ × height^3^ (meters) + *k*_2_ × weight (kilograms) + *k*_3_. *k*_1_ = 0.3669, *k*_2_ = 0.03219, and *k*_3_ = 0.6041 for men; and *k*_1_ = 0.3561, *k*_2_ = 0.03308, and *k*_3_ = 0.1833 for women.

Total red blood cell volume loss = PBV × (Hct_pre_ − Hct_post_), Hct_pre_ = initial preoperative Hct level, Hct_post_ = Hct of third postoperative day.

Total blood loss = 1000 × total red blood cell volume loss/(average of Hct_pre_ and Hct_post_).

Postoperative HB drop = HB_pre_ – HB_post-3_, HB_pre_ = initial preoperative HB level, HB_post-3_ = HB of third postoperative day.

Obvious blood loss = intraoperative blood loss + postoperative blood loss.

Hidden blood loss = total blood loss − obvious blood loss.

#### Statistical methods

All the statistical analyses were performed using IBM SPSS version 19.0 (IBM, USA). Means are presented as mean ± standard deviation (SD), Student’s *t*-test was used to analyze the normally distributed numerical variable; Pearson chi-squared test or Fisher’s exact test was used to analyze the qualitative variable. The significance level used for all tests was *p* < 0 0.05.

## Results

### Patient characteristics

All patients were followed up for 3 months. Detailed distribution of patient demographics and characteristics is presented in Table 1.

**Table 1:** Demographics of both groups

Variable	TXA group (*n* = 39) *n* (%) or mean ± SD (range)	Control group (*n* = 31) *n* (%) or mean ± SD (range)	95% CI (lower to upper)		*p*-Value
			TXA group	Control group	
Age (years)	54.4 ± 13.6 (24–83)	53.7 ± 15.4 (25–83)	50.1–58.6	48.3–59.1	0.841^†^
Sex (%)					0.851^††^
Male	26 (66.7)	20 (64.5)			
Female	13 (33.3)	11(35.5)			
BMI (kg/m^2^)	22.3 ± 2.6 (17.8–25.5)	22.2 ± 2.8 (17.9–28.5)	21.5–23.1	21.2–23.2	0.878^†^
ASA grade (%)	33:6	27:4			0.768^††^
I	33 (84.6)	27 (87.1)			
II	6 (15.4)	4 (12.9)			
HB_pre_* (g/L)	134.2 ± 12.0 (114–159)	134.8 ± 10.1 (113–155)	130.4–138.0	131.2–138.4	0.825^†^

^*^ HB_pre_ = initial preoperative HB level

^†^Student’s *t*-test was used

^††^Chi-squared test was used

### Operative variable

Operative time, intraoperative blood loss, hidden blood loss, total blood loss, preoperative HB level, HB level of the first postoperative day, HB level of the third postoperative day, postoperative HB drop, and transfusion rate are presented in Table 2. The total blood loss, hidden blood loss, and postoperative HB drop in the TXA group were significantly lower than in the control group (*p* < 0.05). There was no statistical difference in terms of operative time, intraoperative blood loss, or transfusion rate between the two groups (*p* > 0.05).

**Table 2:** Clinical outcomes of both groups

Variable	TXA group (*n* = 39) *n* (%) or mean ± SD (range)	Control group (*n* = 31) *n* (%) or mean ± SD (range)	95% CI (lower to upper)		*p*-Value
			TXA group	Control group	
Operative time (min)	57.4 ± 12.8 (43–109)	60.4 ± 11.7 (42–89)	53.4–61.4	56.3–64.5	0.315^†^
Intraoperative blood loss (mL)	106.5 ± 36.1 (78–200)	122.0 ± 32.6 (75–207)	95.5–117.5	111.0–133.0	0.067^†^
Hidden blood loss (mL)	630.5 ± 98.6 (409–807)	893.4 ± 140.3 (644–1175)	599.5–661.5	844.4–942.4	< 0.001^†^
Total blood loss (mL)	736.9 ± 102.2 (567–927)	1015.4 ± 152.4 (773–1285)	704.9–768.9	961.4–1069.4	< 0.001^†^
HB_post-1_^*^ (g/L)	108.9 ± 14.9 (85–140)	99.2 ± 13.8 (80–132)	104.2–113.6	94.3–104.1	0.007^†^
HB_post-3_^*^ (g/L)	87.4 ± 16.3 (63–128)	79.8 ± 10.9 (56–110)	82.3–92.5	76.0–83.6	0.029^†^
Postoperative HB drop (g/L)	46.8 ± 10.8 (22–80)	55.0 ± 13.7 (31–79)	43.4–50.2	50.2–59.8	0.007^†^
Transfusion rate (%)	2.6% (1/39)	12.9% (4/31)			0.279^††^
Postoperative LHS (day)	4.3 ± 0.8 (3–6)	4.6 ± 0.8(3–6)	4.1–4.6	4.3–4.9	0.124^†^
DVT (%)	2.6% (1/39)	3.2% (1/31)			1.000^††^
Harris hip score (point)	91.8 ± 4.9 (80–100)	91.1 ± 6.1 (83–100)	90.3–93.3	89.0–93.2	0.596^†^

^*^ H_pre_ = initial preoperative HB level, HB_post-1_ = HB level of first postoperative day, HB_post-3_ = HB level of third postoperative day, Postoperative HB drop = HB_pre_ – HB_post-3_

^†^Student’s *t*-test was used

^††^Chi-squared test was used

### Clinical results and complications

All patients completed the operation successfully. All patients did not receive blood transfusion on the day of surgery. In the TXA group, 2.6% (1/39) required blood transfusion with 2 units (400 mL) of red blood cell suspension (RBCs) on the third postoperative day, whereas in the control group, 12.9% (4/31) required blood transfusion with 8 units (1600 mL) of RBCs (2 units per patient) (Table 2). There was no statistical difference in terms of postoperative LHS, HHS, or incidence of DVT between the two groups (*p* > 0.05) (Table 2).

Two patients (one patient per group) developed intraoperative fractures of the calcar during seating of the stem, and the fractures were treated by cerclage wire fixation. Subsidence of the stem during loading was not observed after 3 months of follow-up. Two patients (one in each group) had asymptomatic DVT and did not receive any special treatment. No incisional infection occurred in either group. No patient died during the study period.

## Discussion

There is growing evidence that TXA is effective in reducing blood loss and transfusion rates in the perioperative period of THA. Most studies have been performed using the posterolateral approach [12, 18] or the lateral approach [19] or the posterior approach [20, 21]. Although some studies [13, 14, 22–24] have reported the efficacy of TXA in DAA, few studies have compared the efficacy and safety of DAA-THA with and without TXA [13, 14]. This study aimed to explore whether TXA reduced perioperative blood loss and the rate of blood transfusion in patients undergoing THA via the DAA.

In the present study, 1.5 g of TXA was infused intravenously, and it was effective in reducing total blood loss, hidden blood loss, and the degree of Hb drop, but had no significant effect on intraoperative blood loss or transfusion rate during the perioperative period in DAA THA. Fraval et al. [13] performed a single-center randomized, double-blind trial of 101 patients undergoing THA via DAA, 50 of whom received TXA during the perioperative period. The results found that TXA significantly reduced the blood loss (both intraoperative and calculated blood loss), but there was no statistical significance in transfusion rate between the both groups. Our results are consistent with those of Fraval et al. [13], except for the intraoperative blood loss. We speculate that the reason for this is that the patients in our study were younger and had a lower BMI and shorter operative time than those in the study by Fraval et al. [13], because these factors would theoretically reduce intraoperative blood loss (our patients: 106 mL; Fraval’s patients: 460 mL). In addition, our findings are identical to those of Free et al. [14], who found a postoperative transfusion rate of 1.2% for THA with DAA, which was significantly lower than the 11.1% transfusion rate in the control group. We speculate that the reason for this is that (1) our patients were younger and have better tolerance to blood loss, so there is no difference in blood transfusion rates between the two groups (TXA group versus control group), while Free's patients were older and may have poor tolerance to blood loss, so the blood transfusion rates between the two groups (DAA without TXA versus DAA TXA) were different; (2) the sample size of these patients in our study was smaller than that of the patients in the study by Free et al. [14]. Whether TXA can reduce the transfusion rate for THA via DAA requires a larger sample size and more prospective studies to determine.

Few studies [13, 14] have found that the use of TXA during the perioperative period can reduce the LHS in THA. However, our study found that the use of TXA during the perioperative period of THA via DAA did not shorten the postoperative LHS. We speculate that this is because DAA is a minimally invasive procedure that shortens the LHS. Whether TXA shortens LHS for THA via DAA requires a larger sample size and more prospective studies to determine.

Many studies [12, 25, 26] have reported that the use of TXA did not increase the incidence of DVT in patients undergoing THA. However, Nishihara et al. [27] conducted a study to observe whether TXA increased the risk of DVT in lower limbs without routine chemical thromboprophylaxis, and found that the use of TXA increased the incidence of distal DVTs in the muscular veins. Our study found one case of DVT in the TXA group, and one case in the control group, with no statistically significant difference (*p* = 1.000).

There are several limitations to this study. The incidence of DVT in the lower extremities was assessed only in the short term. Our sample size was small, and the results may have been biased. A larger randomized prospective trial is required to further improve the relevant experiments to determine the efficacy and safety of TXA in the perioperative period of THA via DAA.

## Conclusion

Single intravenous administration of 1.5 g of TXA 10 min prior to incision may effectively reduce the perioperative blood loss in primary THA through DAA, without increasing the incidence of DVT in lower extremities.

## Data Availability

The datasets used or analyzed during the current study are available from the corresponding author on reasonable request.

## Abbreviations

TXA: Tranexamic acid
THA: Total hip arthroplasty
DAA: Direct anterior approach
HB: Hemoglobin
DVT: Deep vein thrombosis
LHS: Length of hospital stays
HHS: Harris hip score
BMI: Body mass index
HCT: Hematocrit
ASA: American Society of Anesthesiologists
PBV: Pulmonary blood volume
